# A platform for soybean molecular breeding: the utilization of core collections for food security

**DOI:** 10.1007/s11103-013-0076-6

**Published:** 2013-05-25

**Authors:** Li-Juan Qiu, Li-Li Xing, Yong Guo, Jun Wang, Scott A. Jackson, Ru-Zhen Chang

**Affiliations:** 1 The National Key Facility for Crop Gene Resources and Genetic Improvement (NFCRI), Institute of Crop Science, Chinese Academy of Agricultural Sciences, Beijing, 100081 China; 2 Center for Applied Genetic Technology, University of Georgia, Athens, GA 30602 USA

**Keywords:** Plant genetic resources, Soybean, Utilization of core collection, Molecular breeding, Food security

## Abstract

Soybean is an important crop not only for human consumption but also for its addition of nitrogen to the soil during crop rotation. China has the largest collection of cultivated soybeans (*Glycine max*) and wild soybeans (*Glycine soja*) all over the world. The platform of soybean core, mini core and integrated applied core collections has been developed in the past decade based on systematic researches which included the sampling strategies, statistical methods, phenotypic data and SSR markers. Meanwhile, intergrated applied core collections including accessions with single or integrated favorite traits are being developed in order to meet the demand of soybean breeding. These kinds of core collections provide powerful materials for evaluation of germplasm, identification of trait-specific accessions, gene discovery, allele mining, genomic study, maker development, and molecular breeding. Some successful cases have proved the usefulness and efficiency of this platform. The platform is helpful for enhancing utilization of soybean genetic resources in sustainable crop improvement for food security. The efficient utilization of this platform in the future is relying on accurate phenotyping methods, abundant functional markers, high-throughput genotyping platforms, and effective breeding programs.

## Introduction

Food security exists when all people, at all times, have physical, social and economic access to sufficient, safe and nutritious food (FAO 2003). The sufficiency of human food is dependent in large on the availability and nutrient sufficiency of the plants consumed directly, or indirectly through animals (Bruulsema et al. 2012). Of the 7,000 plant species used worldwide in food and agriculture, only 30 crops ‘feed the world’, which provide 95 % of global plant-derived energy-intake (calories) and proteins (Schmidt and Wei 2006). The soybean is a crop of global importance and is one of the most frequently cultivated crops worldwide. It is important for both protein meal and vegetable oil and is used for both human and animal consumption as well as for industrial purposes, such as biofuels (Hartman et al. 2011). In addition, the soybean also plays an important role in crop diversification and benefits other crops due to its addition of nitrogen to the soil during crop rotation (Singh 2010). Soybean seeds contain about 20 % oil and 40 % protein. Of the oil fraction, 95 % is consumed as edible oil with the rest used for industrial products from cosmetics and hygiene products to paint removers and plastics. In addition, about 98 % of the soybean meal is used in livestock and aquaculture feeds due to its high protein level (Liu 2008). Soybeans are unique among crop plants in that they supply protein equal in quality to that of animal sources but with less saturated fat and no cholesterol (Young 1991). For this reason, soybeans have long been consumed in Asia and greatly increased in popularity outside of Asia as a primary source of protein in such traditional foods as tofu, soymilk, tempeh, natto, sprouts, green vegetable soybeans, and many others (www.soyinfocenter.com).

**Fig. 1:**
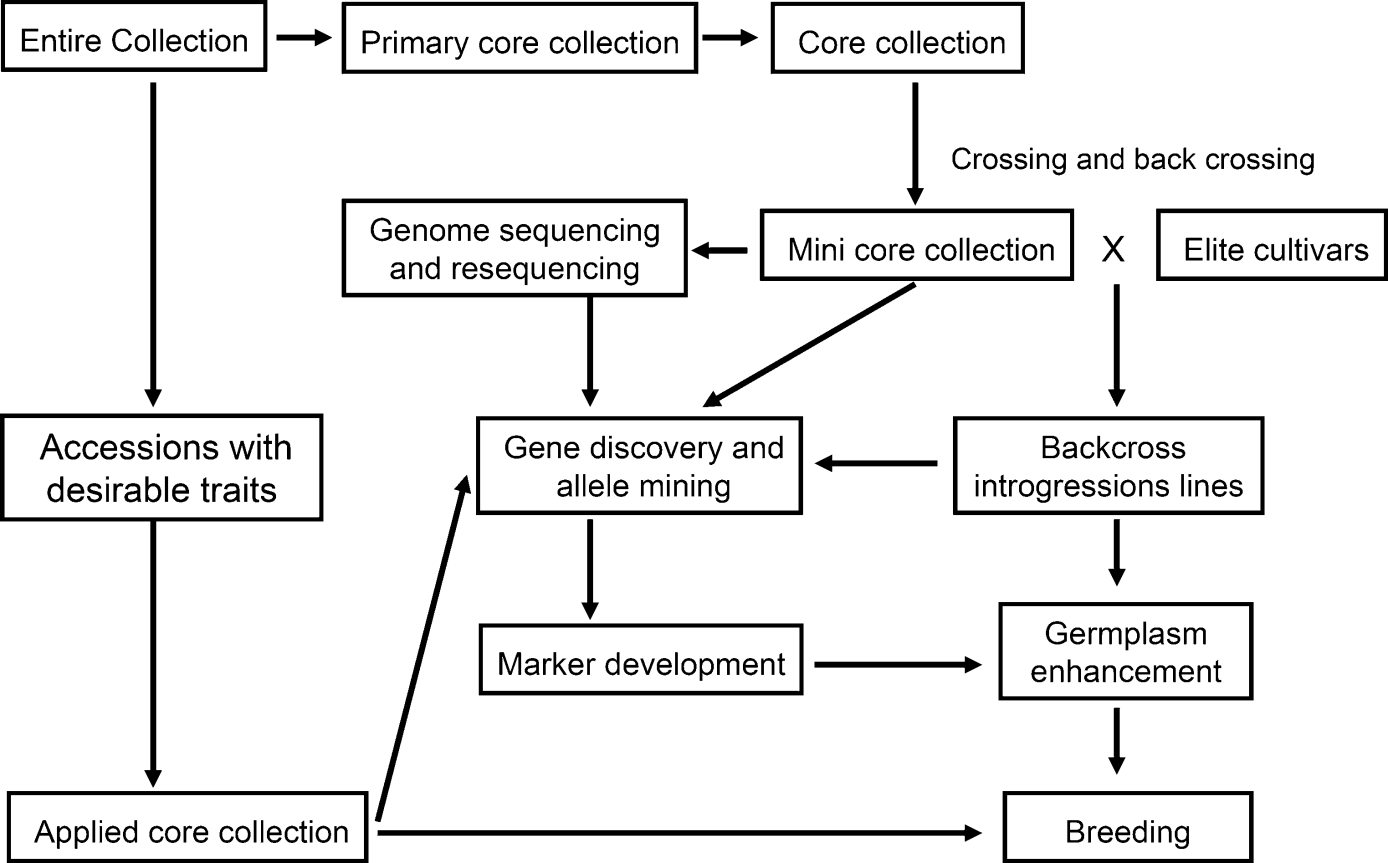
The development and utilization of soybean core collections

Soybean was domesticated in China about 4,500 years ago, during the ancient Huangdi period (Qiu et al. 2011a). By the 16th century A.D. soybean was transported to Japan, Indonesia, Philippines and Vietnam, and later introduced to Europe and America (Hymowitz and Newell 1980). It is now cultivated in more than 60 countries across five continents. As the center of cultivated soybean (*Glycine max*), China has the most abundant genetic resources for soybean (>23,000 accessions) and its wild relatives, *Glycine soja* (>7,000 accessions). Chinese soybean germplasm is used widely throughout the world and these accessions are useful for small farmers that have to cope with heterogeneous microclimates and for advanced soybean improvement programs and to deal with needs in the future such as climate change. Although a large number of soybean accessions are now conserved either in various genebanks or in situ, <1 % of them have been used for breeding. In order to increase the efficiency of utilization of soybean genetic resources, different types of core collections have been developed from the whole collection of Chinese soybean germplasm, using a combination of passport data and morphological traits (Qiu et al. 2003; Song et al. 2010; Wang et al. 2006; Zhao et al. 2005). In this paper, the development and utilization of different core collections for Chinese soybean germplasm as a platform were reviewed. This platform is helpful for enhancing utilization of soybean genetic resources for sustainable crop improvement for food security (Fig. 1). The challenges and prospects of this platform for molecular breeding and food security were also discussed.

## Development of soybean core collections

Plant germplasm is the lifeblood of plant breeding without which breeding is impossible to conduct. It is important that the diversity within major crops not only conserved but managed wisely and mined for useful traits. Although these abundant resources provide a rich genetic base for breeding, they also bring the difficulties for conservation and utilization. The approaches of core collection and mini core collection provided a rational approach to use a manageable number of accessions from large germplasm collections (Brown 1989; Frankel and Brown 1984; Upadhyaya and Oritz 2001). A core collection is a subset of accessions that represents the genetic diversity of a species and its relatives. Those samples not included in the core collections are not abandoned, but rather maintained as “reserved collection”, the total number of resources referred here is over 3,000 accessions, take 10 % core collections represent 70 % of the whole collection (Frankel and Brown 1984). A mini core collection consists of 10 % accessions of the core collection, and hence only 1 % of the entire collection (Upadhyaya and Oritz 2001). The mini core collection still represents the diversity of the entire collection but the number is largely reduced compared with that of core collection. Due to the reduced size, core collection and mini core collection could be studied extensively and the information derived can be used to guide more efficient utilization of the much larger reserved collection. Up to now, more than 35 core collections and mini core collections have been developed for nearly 30 species, including rice, wheat, maize, soybean, chickpea, peanut, common bean, and so on (Balfourier et al. 2007; Blair et al. 2009; Hao et al. 2008; Holbrook and Dong 2005; Li et al. 2004; Upadhyaya et al. 2001; Upadhyaya and Oritz 2001; Wang et al. 2006; Zhang et al. 2011a).

In soybean, the first core collection was developed in 1987 based on the passport data but without any follow-up reports (Brown et al. 1987). Since then, core collections of cultivated and wild soybean were developed based on different gene pools all over the world. A core collection of soybean accessions was used for analysis of the distribution of the major soybean seed allergens (Yaklich et al. 1999). The Chinese cultivated soybean core collection has been developed from 23,587 accessions conserved in the Chinese National Soybean GeneBank (CNSGB) (Wang et al. 2006) and 652 accessions were selected to represent the total 6,172 accessions of Chinese anneal wild soybean (Zhao et al. 2005). A core set of Korean landraces was developed from approximately 7,000 accessions conserved in the National Genebank of Rural Development Administration, Korea (Cho et al. 2008). A core collection (1,600 accessions) was also selected from 16,999 accessions in USDA Soybean Germplasm Collection (Oliveira et al. 2010). Recently, based on passport data records, a Japanese landrace subset consisting of 832 accessions was proportionally selected from 3,994 accessions of the National Institute of Agrobiological Sciences Genebank (Kaga et al. 2012). Among all these core collections of soybean, Chinese soybean core collection (2,794 accessions of cultivated soybeans and 652 accessions of wild soybeans) was the largest and systemically studied one.

### The strategy for development of soybean core collections

The strategy for development of soybean core collections included the following aspects. First, the samples which were mainly domestic soybean accessions supplemented with exotic germplasm to broaden the diversity within the core. Second, data acquisition was based on passport data, taking into consideration of agronomic traits and molecular markers. Third, the selection was based on stratification and statistics as well as experienced artificial selection. The representativeness was tested on primary core collection (Cui et al. 2003, 2004), core collection (Wang et al. 2006) and mini core collection (Wang et al. 2008) levels with different criteria (Qiu et al. 2009). Therefore, the soybean core and mini core collections were developed through selection, test, supplement, retest, and systematic representativeness analyses. In addition, single and integrated applied core collections were also developed with desirable traits based on breeding demand. The characteristic of all this collections were broaden representative, high diversity but meet different demand of study and breeding.

### From entire germplasm collection to core and mini core collections

More than 23,000 cultivated soybean accessions and 7,000 wild soybean accessions are conserved in the CNSGB now. These accessions include local landraces and wild soybeans collected in China and overseas, improved varieties and breeding lines developed by Chinese soybean breeders or introduced from overseas agricultural research institutes. In order to detect the redundancy of these accessions, some accessions including six accessions with the same name “Man cang jin” were characterized by agronomic traits and SSR markers. The results showed that all of these accessions had obvious difference at both agronomic and molecular level, indicating the extremely low redundancy of accessions stored in CNSGB (Yan et al. 2003). In order to select proper core collection of cultivated soybean for further molecular evaluation, 20 sampling methods were compared using passport data and agronomic traits, and the optimal strategy and sample size were selected (Qiu et al. 2003). The samples were analyzed with core set of SSR markers that were selected and tested using small amount of samples (Wang et al. 2003; Xie et al. 2003). By integration of the SSR marker data and agronomic traits, a candidate core collection of cultivated soybean was developed. Then the selected candidate core collection was supplemented with additional 130 accessions with various agronomic traits and 494 accessions with disease resistances and stress tolerance (Wang et al. 2003, 2006). The core collection of Chinese cultivated soybean consisting of 2,794 accessions was developed, which accounted for 11.8 % representative of the entire collection and 73.6 % of genetic diversity separately (Qiu et al. 2009). At the same time, core collection of annual Chinese wild soybean was also developed by comparing five sampling strategies with genetic representation of 83.6 % for the entire collection (Zhao et al. 2005).

In order to easy evaluation of soybean core accessions for replication at different locations, mini core collections of soybean were also developed due to the large size of entire collection of soybean. The mini core collection of cultivated soybean consisting of 248 accessions was developed based on the primary core collection and it represented 94.5 % of the phenotypic diversity and 63.5 % of the genetic diversity respectively (Qiu et al. 2009; Wang et al. 2006). SSR marker analysis also showed that this mini core collection had high genetic diversity (Song et al. 2010). Furthermore, a set of mini core collection of 96 wild soybean accessions were also evaluated for the extent of genetic diversity (Liu et al. 2009).

### From trait-specific core collection to integrated applied core collection

The trait-specific accessions in core collection and mini core collection were scare due to the need of including as many accessions with various traits under the premise of the appropriate sample size as possible. Therefore, the elite germplasm identified in the mini core collection could only be used as an indicator for directional identification of entire collection. Since soybean is a short-day plant and it adapts to a narrow geographic range, accessions adapted certain geography would have difficulties in adapting to varied eco-regions in breeding, especially for yield-related traits. Therefore, selecting more accessions with the same desirable traits for evaluation will be valuable for soybean improvement. The concept of applied core collection concerned representative germplasm with targeted desirable traits and high level of genetic diversity. Applied core collection of soybean could fulfill the needs of accessions adapted different eco-regions and broadening the genetic base of breeding programs. Up to know, different desirable traits, including resistance to soybean cyst nematode (SCN) (Duan et al. 2008; Ma et al. 2006), resistance to soybean mosaic virus (SMV) (Mi et al. 2004), high phosphorus efficiency (Zhao et al. 2004) etc., have been used to develop applied core collections of soybean. These core collections all encompassed more than 70 % of the genetic variation present in the overall collection with targeted traits.

However, the sample size would increase suddenly if developing one applied core collection for each specific desirable trait, which would be inconvenient for research and utilization and go against the principle of core collection. Therefore, soybean integrated applied core collection consisting of accessions with cold tolerance, drought tolerance, salt tolerance, resistance to SCN, resistance to SMV, high protein content, and high fat content was developed (Guo et al. 2013). These accessions showed a high level of diversity in terms of target traits, non-target traits and molecular markers. Meanwhile, the integrated applied core collection of soybean not only had increased the number of soybean accessions with specific desirable traits but also had similar diversity as mini core collection. Therefore, the concept of integrated applied core collection resolves the conflict between reducing sample size and concentrating genetic diversity and solves the problem of establishing many core collections for different traits. Accessions with integrated excellent traits in breeding would be conducive in improving varieties and shortening the time for application in breeding.

## Utilization of core collections: from phenotypes to genes/markers and breeding

Due to the proper sample size, high diversity, and broad representative, core collections are ideal resources for phenotypic, genetic, genomic researches and breeding program. After development of different kinds of core collection, the utilization of them have become more and more important. As a model crop, rice research is always ahead of other crops including wheat, maize, soybean, and so on. Many important genes including *GS3*, *PROG1*, *IPA1*, *DTH8*, and others, were cloned successfully using introgression lines (ILs) or near-isogenic lines (NILs) based on germplasm collections of rice (Fan et al. 2006; Jiao et al. 2010; Jin et al. 2008; Tan et al. 2008; Wei et al. 2010). Using 21 elite varieties of rice as recurrent parents, and 188 donor parents originating from 24 countries or regions, approximately 60,000 ILs and over 17,000 NILs based on the mini core collection of rice have been developed systemically (Li et al. 2011; Long et al. 2009). Similarly, soybean core collections have been played and will play an important role in basic research of soybean biology and soybean breeding for food security.

### Germplasm evaluation and identification

The core and mini core collections of the germplasm were evaluated at diverse locations to identify trait-specific diverse accessions. Due to the reduced size, the core and the mini core collections have been characterized more precisely and some very useful trait specific accessions have been identified (Upadhyaya et al. 2008). These accessions are similar to or better than the control cultivar(s) for a particular trait, agronomically good and genetically diverse. Some plant germplasm with desirable agronomic and nutritional traits had been identified using core collection and mini core collection, such as chickpea with drought-avoidance root traits (Kashiwagi et al. 2005), pigeonpea with drought resistance (Upadhyaya et al. 2007), pigeonpea with resistance to multiple disease (Sharma et al. 2012), peanut with high-quality (Chu et al. 2007) and wheat with high-molecular-weight glutenin subunits (Zhang et al. 2002). Soybean core collection and mini core collections also played an important role in the identification of desirable agronomic and nutritional traits. Although only part of the core collections was used in traits identification, variation was observed and extensively existed for both yield and quality related traits by testing on one site or multi-sites (Table 1). For example, there was threefold variation in the amount of allergic protein 28 K (Zhang et al. 2006) to over 12-fold for protein subunit (Wang et al. 2008). Except seed quality and yield, the variations were also observed for identifying the disease resistance. Using four SMV strains to test 99 accessions in soybean core collections from Huanghuai and South part of China, accessions with all six resistant levels were identified (Chen et al. 2009). One accession was resistant to the four strains, three accessions were resistant to three strains except SC-7, and six accessions with ability to keep disease lesion from spreading, all of which are valuable in breeding (Chen et al. 2009). The identification of these trait-specific diverse accessions also proved that core collections were representative of the larger collection and adequately captured useful genetic variation.

**Table 1:** Summary of desirable traits evaluation with core or mini core collections in soybean

Traits	Germplasm		Evaluation			References
	Collection	Number	Unit	Average	Range	
Quality						
28 K allergen deficient	Core	220	%	33	21.1–61.5	Zhang et al. (2006)
11S/7S	Mini core	205	Ratio	1. 56	0. 55–4. 95	Wang et al. (2008)
Isoflavone	Mini core	100	mg/g	3.68	0.62–7.66	Wang et al. (2011a)
Isoflavone	Core	300	mg/g	2.39	0.18–6.07	Yuan et al. (2011)
α′ subunit	Mini core	236	%	8.7	0.2–16.7	Jian et al. (2012)
α subunit	Mini core	236	%	5.4	0.5–13.1	Jian et al. (2012)
β subunit	Mini core	236	%	15.0	5.8–29.3	Jian et al. (2012)
A3 subunit	Mini core	236	%	7.8	3.0–15.3	Jian et al. (2012)
A1 A2 A4 subunit	Mini core	236	%	22.8	13.4–32.0	Jian et al. (2012)
B subunit	Mini core	236	%	40.4	20.5–52.6	Jian et al. (2012)
11S/7S	Mini core	236	Ratio	2.94	1.228–13.745	Jian et al. (2012)
Yield						
Plant height	Mini core	60	cm	–	65.9–154.83	Liu et al. (2011)
Branches	Mini core	60	No.	–	0.85–5.16	Liu et al. (2011)
100-seed weight	Mini core	60	g	–	8.96–25.65	Liu et al. (2011)
Seed weight	Mini core	60	kg/hm	–	1,647.35–3,070.07	Liu et al. (2011)
Plant height	Core	195	cm	89.9	24.0–263.4	Hu et al. (2009)
Effective branches	Core	195	No.	4.2	0–12.0	Hu et al. (2009)
100-seed weight	Core	195	g	13.4	4.3–25.3	Hu et al. (2009)
Growth duration	Core	195	day	94.8	71–125	Hu et al. (2009)
Nodes on main stem	Core	195	No.	19.1	9.5–29.4	Hu et al. (2009)
Effective pods per plant	Core	195	No.	110.4	22.8–333.0	Hu et al. (2009)
Total pods per plant	Core	195	No.	119.2	25.3–338	Hu et al. (2009)
Pods per node	Core	195	No.	6.3	1.5–15.1	Hu et al. (2009)
Podding height	Core	195	cm	9.3	0–28.2	Hu et al. (2009)
Seed length	Mini core	235	mm	7.5	4.8–10.5	Wang et al. (2011b)
Seed width	Mini core	235	mm	5.9	3.4–8.5	Wang et al. (2011b)
Seed thickness	Mini core	235	mm	4.4	1.9–7.2	Wang et al. (2011b)
100-seed weight	Mini core	235	g	15.1	4.84–34.21	Wang et al. (2011b)

### Gene discovery and allele mining

Due to abundant in diversity and practical in scale, core collections and mini core collections could be used for gene discovery and allele mining (Table 2). One way for gene discovery using core collection is association analysis of core collections and their derived lines. With the development of new sequencing technology and availability of core collections, it is now feasible to establish an open-source translational research platform for genome-wide association studies (GWAS). GWAS have contributed tremendously to discovering genes controlling natural variations of complex traits in rice (Huang et al. 2010, 2012). Using SSR and SNP markers to genotype the core collections of soybean, linkage disequilibrium and association for plant height, maturity, 100-seed weight, protein content and oil content were also carried out. Twenty-one SSR markers on 16 chromosomes were found significantly (*P* < 0.01) associated with these traits (Li et al. 2010). Genome-wide association analysis of 191 accessions based on the unified mixed model also identified 19 SNPs and five haplotypes associated with soybean yield and yield components in three or more environments (Hao et al. 2012).

**Table 2:** Marker-trait association by directly and indirectly using core collections in soybean

Name of sample	No. of sample	Trait	No. of QTLs	References
*Core collections*				
Partial mini core collection	189	Yield and yield component	19	Hao et al. (2012)
Partial mini core collection and intergated applied core colletion	159	High oil content	6	Li et al. (2010)
		High protein content	1	
		Drought tolerance	5	
		SCN resistance	6	
		SMV resistance	3	
*Backcrossing lines*				
Backcrossing lines of Clark with Hongfeng 11	46	Low temperature resistant	13	Jiang et al. (2009)
Backcrossing lines of Clark with Hongfeng 11	72	Salt tolerance	3	Li et al. (2009)
		Drought tolerance	5	
		Agronomic	17	
Backcrossing lines of Clark with Hongfeng 11	23	Salt tolerance germination stage	22	Qiu et al. (2011b)
		Low temperature tolerance	15	

The other powerful tool to explore target genes controlling important traits is applying ILs and NILs developed from core collections. In a random backcross population, the allelic and genotypic frequencies at a given locus are known. A significant deviation of donor allele frequency at a single locus in ILs from the expected Mendelian level implied a positive selection favoring the donor allele and inferred a possible putative QTL locus. In soybean, ILs and NILs also becoming important to isolate genes controlling important traits such as photoperiodic flowering, leaflet shape and number of seeds per pod (Jeong et al. 2012; Xia et al. 2012). QTLs related to cold and drought stress had also been identified by using backcross introgression lines developed from mini core collection (Jiang et al. 2009; Qiu et al. 2011b).

Plant accessions from wild or locally adapted landrace genepools conserved in genebanks contain a rich repertoire of alleles that have been left behind by the selective processes of domestication, selection and cross-breeding that paved the way to today’s elite cultivars (Kilian and Graner 2012). Most of accession in core collection and mini core collection of cultivated and wild soybean are either wild or landrace. The mini core collection or the reference sets (subset of mini core) could constitute the starting materials for dissecting the naturally occurring allelic variation at candidate genes through allele mining efforts (Kumar et al. 2010). Mini core collection of soybean was used to identify allele distribution of *GmTFL1* gene, which regulated the determinate growth habit in soybean. The finding that four *Gmtfl1* alleles were observed among cultivated soybeans whereas wild soybeans only contained one *GmTfl1* allele reflected the effects of genetic bottlenecks created by soybean germplasm introduction and modern breeding (Tian et al. 2010). Three low frequency new alleles of *GmF3′H* and *GmF3′5′H* were also identified using partial mini core collection, which indicated that the materials used for genotyping had abundant genetic variation (Guo and Qiu 2013).

### Genomic study and marker development

With the advent of reference genome sequences for many crops, genomic approaches can be used to find specific genes and alleles and to clarify the origin, differentiation, domestication, and genome evolution of crops. Completion of the soybean genome sequence opens a new and exciting chapter for soybean biology study (Schmutz et al. 2010). A total of 17 wild and 14 cultivated soybean genomes were re-sequenced to an average of approximately ×5 depth and >90 % coverage. A set of 205,614 tag SNPs that may be useful for QTL mapping, association studies and marker development were identified (Lam et al. 2010). A subset of 25 accessions from the mini core collection were also used for resequencing and about 25.5 % SNPs were novel SNPs when compared them with the resequencing data of 31 accessions because the former set of accessions has more genetic diversity than the later set of acceesions (Li et al. 2013). Furthermore, re-sequencing of wild soybean *G. soja* and a subsequent comparative genomic analysis with reference to the *G. max* genome also identified a large degree of nucleotide and structural variation between the wild and domesticated soybean (Kim et al. 2010). The variation identified by resequencing of soybean germplasm could be used for marker development.

### Molecular breeding and germplasm enhancement

With advances in identification of QTLs/genes responsible for important agronomic traits, molecular breeding is gradually becoming an actuality. Molecular markers developed from functional genes were used to introgression soybean cultivars for improving important agronomic traits, such as yield, quality, disease resistance and tolerance abiotic stresses. Molecular breeding has been used in breeding of SCN-resistance cultivar (Zhonghuang 57), cultivar with *Lox2*-deficient (Zhonghuang 18), cultivar with trypsin inhibitor-deficient (Zhongdou 28), and cultivar with double deficient of *Lox2* and *Lox3* (Wuxing No.1) .

Most of local varieties/landraces in soybean core collections are no longer used for production and in breeding. In order to better exploit the important agronomic traits of these germplasm in soybean breeding, backcross introgression lines were made by cross and backcross of advanced varieties with mini core collections, which could broaden the genetic base of soybean and conduct germplasm enhancement for improvement. Since most of landraces usually have undesirable traits such as low yield, late maturity and easy lodging, they are difficulty to be used for genetic improvement via traditional breeding. Backcrossing can shorten the time to achieve improved varieties by taking superior yielding varieties and introgressing new traits. The contribution analysis of the one accessions to its derivatives showed that more than 77 % of the varieties derived from soybean accession Tokachi-Nagaha had <25 % genetic base of this accession (Guo et al. 2007). This indicated that multiple crossing or backcrossed could eliminate undesirable traits and have good adaptability. Recent studies showed that backcrossing twice using modern varieties as receptor crossing with accessions from mini core collection, most of the undesirable traits could be improved remarkably (Yan et al. 2012; Zhang et al. 2011b). It was found that BC_2_F_2_ progenies had higher protein content that was positively correlated to pods per plant, kernels per plant and 100-seed weight. Especially, the yield potential of lines developed by backcrossing the mini core collections appeared to be promising. This strategy of breeding, based on the backcrossing of modern cultivar with accessions from core collections, is significant in sustainable maintaining and developing useful variation and providing new genes to improve soybean cultivars continuously.

## Challenges and prospects

Considering the huge number of accessions that are held collectively in various gene banks, genetic resources collections are deemed to harbor a wealth of undisclosed allelic variants. The rich genetic diversity of plant genetic resources is a prerequisite for germplasm improvement for food security. The systematic study of soybean core collections also provides a platform for soybean molecular breeding in the future. Now the challenge is how to efficiently identify and exploit the useful genes, alleles, molecular markers and variation for cultivar improvement.

### Accurate phenotyping methods

Phenotyping would be the key for success as phenotypes are considered as the best clues for genotypes. Increase in precision of phenotyping techniques maximizes the chances of ‘mining’ the prospective genotypes. Also, accurate evaluation of phenotypes of soybean core collations in different regions with duplication will accelerate the course of gene discovery and marker development. Hence, there is a need to refine phenotyping protocols and equipments to increase the efficiency of phenotyping.

### Abundant functional genes and markers

Identification of functional genes and markers is the base of molecular breeding. More and more functional genes will be isolated and functional markers will be developed by using the soybean germplasm resources, which also rely on nucleotide and/or structural variation on whole genomic level. Moreover, wild relatives of crops are a reservoir of genetic variation that can be utilized to increase yield and response different stress. Therefore, it is necessary to integrate genomes of a set of representative individuals (pan-genome) by de novo sequencing wild/cultivated soybeans and comparison of the reference genome.

### High-throughput genotyping platforms

As technology advances and the cost drops, next-generation sequencing technologies revolutionized our ways on germplasm characterization. Accessions from core collections will be able to be genotyped with large number of markers or even sequenced at whole genome level in the foreseeable future. High-throughput genotyping platforms will be used for effective genome-wide gene-trait association studies to identify genes useful for genetic improving crop productivity.

### Effective breeding programs

Molecular markers of single gene/locus could be used for molecular breeding in soybean. The advances in soybean research and the enrichment of large-scale marker data sets provide us tools to determine the genetic basis of all important agronomic traits. Based on genes uncovered for these traits, breeders can design and combine all the most favorable alleles into one variety, and then use molecular markers closely linked to these loci to develop the desired varieties. Meanwhile, breeding genetically modified soybean using favorable genes identified from soybean germplasm resources will also be another choise for molecular breeding. The molecular design breeding will play an important role in soybean breeding for food security.
